# Assessing the operational effectiveness of a maternal and child health (MCH) conditional cash transfer pilot programme in Nigeria

**DOI:** 10.1186/s12884-019-2418-0

**Published:** 2019-08-16

**Authors:** Chioma Oduenyi, Victor Ordu, Ugo Okoli

**Affiliations:** 1 Jhpiego, Nigeria-affiliate of Johns Hopkins University, Abuja, Nigeria; 2 Global Health and Medical Consultants Limited, Abuja, Nigeria; 3 SURE-P MCH Project Implementation Unit, National Primary Health Care Development Agency, Abuja, Nigeria; 4 National Environmental Standards and Regulations Enforcement Agency, Abuja, Nigeria

**Keywords:** SURE-P MCH, Conditional cash transfer, Pilot, Operational processes, Effectiveness, SWOT analysis, Challenges, Recommendations

## Abstract

**Background:**

This paper provides insights into design and implementation of a Conditional Cash Transfer (CCT) pilot programme under the Subsidy Reinvestment and Empowerment Programme on Maternal and Child Health (SURE-P MCH) in Nigeria. The CCT day to day operations were independently assessed, from design to enrollment and pay out, in order to inform future CCT designs and implementation.

**Methods:**

This study combined a desk review of SURE-P MCH CCT operational documents and retrospective, descriptive cross-sectional survey of 314 primary beneficiaries of the CCT scheme from 29 SURE-P MCH CCT designated health facilities between June – July 2015. The programme implementation manual (PIM) and several CCT monthly reports and articles obtained from the project implementation unit (PIU) were reviewed while structured questionnaire of (16) questions was used for face-to-face interviews with (30–33) CCT beneficiaries drawn from each of eight (8) participating states of Anambra, Bauchi, Bayelsa, Ebonyi, Kaduna, Niger, Ogun, and Zamfara and the Federal Capital Territory (FCT)-Abuja. Findings were analyzed and reported using R* statistical package (version 3.1.2). Subsequently a strengths, weaknesses, opportunities and threats (SWOT) analysis was conducted to identify key challenges and possible recommendations.

**Results:**

The SWOT analysis indicated a robust design for the CCT programme, which would have enhanced operational effectiveness if implemented as designed. However, the programme faced several implementation challenges. For instance, though 65% of beneficiaries perceived CCT pay-out events to be orderly and well-organized, in some of the pilot states the events were marred with inconsistencies resulting in large crowds and increased waiting time for some beneficiaries. Similarly, only 40% of beneficiaries received the complete N5,000 (USD30) cash incentive, 28% received N1,000 (USD6) while others received either N2000 (USD12), N3000 (USD18) or N4000 (USD24).

**Conclusion:**

The CCT pilot had a robust design as a result of a successful proof of concept which preceded the pilot roll-out. However, its implementation was marred with several challenges ranging from untimely release of funds, limited monitoring and evaluation and other operational challenges. Future CCT programmes should understudy the SWOT analysis presented in this paper to improve the design and implementation of CCT programmes in Nigeria and other settings.

## Background

Nigeria’s high maternal mortality of 576 per 100,000 live births and infant mortality of 70 per 1000 live births [1, 2] remain worrisome and reflects inequality in maternal and infant mortality due to poverty. Hence one strategy to fight health inequality due to poverty is conditional cash transfers (CCTs) [3]. Obviously, the last two decades have seen an upsurge in the use of conditional cash transfers (CCTs) as innovative approaches to deliver social goods to vulnerable populations around the world [4–6]. Since, Mexico implemented *Oportunidades* and Brazil’s *Bolsa Familia Programme* (BFP), several countries have implemented CCTs from Latin Americas to Asia such as the Janani Suraksha Yojana (JSY) CCT programme in India [7] and Indonesia’s large-scale CCTs scheme known as Program Keluarga Harapan (PKH) [3, 8]. Sub-saharan Africa such as Kenya has also implemented CCTs and most recently in developed countries such as England and “Opportunity New York City” (ONYC) in the United States [9–12]. Obviously, most CCTs are targeted at disadvantaged or vulnerable people as investments in human capital and sometimes, providing immediate poverty relief [13].

As a lead social protection initiative in addressing poverty and vulnerability, CCT is not totally new to Nigeria. Nigeria has had its own share of CCT schemes at different scales and with varied focus since the 2000s and while some were state-sponsored, others were federal government-sponsored. Prominent among CCTs in Nigeria was the “In Care of the People” (COPE) conditional cash transfer (CCT) programme launched in 2007 across 12 Nigeria’s states, targeted at reducing socio-economic vulnerabilities and breaking the cycle of intergenerational poverty [14]. The effectiveness of COPE became questionable when monitoring and evaluation (M&E) mechanisms were conspicuously lacking and many challenges with the programme delivery and infrastructure which undermined the programme’s effectiveness [14]. Other previous CCTs programmes include; CCTs to support girl-child education in three Northern states; Kano, Bauchi and Katsina assisted by the World Bank, DFID and UNICEF. Also, Jigawa state sponsored a disability allowance programme while Bayelsa state sponsored a child savings scheme. Cross -Rivers State also sponsored a CCT programme for poor households and Ekiti State targeted the elderly who do not earn pensions but were above 65 years (though unconditionally) [14].

Unfortunately, these schemes have been largely unsustainable because they have been characterized by poor conceptualization and short-term financing mechanisms which usually lasted between 1 and 2 years’ timeframe. The schemes also lacked institutional capacity in terms of robust guiding policies, effective M&E systems and inter-sectoral coordination. Consequently, due to the absence of these accountability and transparency mechanisms, the effectiveness of the CCT initiatives as an instrument of social protection in Nigeria has been questionable [14, 15]. Despite challenges of previous CCTs programmes in Nigeria, the overwhelming evidence around positive outcomes of CCT schemes around the world [16] motivated the Federal Government of Nigeria (FGN) to implement SURE-P MCH CCT. However, the CCT pilot programme which started in April 2013 ended in May 2015 following a democratic transition to a new government regime and gains made by the project may have been reversed within a short time with the discontinuation of the scheme.

The FGN initiated the SURE-P maternal and child health programme (SURE-P MCH) as a combination of supply and demand-side interventions. The scheme was initially launched in 500 primary health care (PHCs) facilities across Nigeria’s 36 states and the Federal Capital Territory (FCT) and later scaled to 1000 facilities [17]. As part of the demand –side intervention, a six-month proof of concept phase was implemented in two area councils in the FCT (Kuje and Bwari) in 2012 to test the CCT implementation design. Consequently, upon a successful completion of the proof of concept phase, the CCT component was piloted to increase demand for basic maternal, newborn and child health (MNCH) services and not necessarily for poverty reduction as earlier CCT schemes targeted [18]. The CCT pilot scheme was implemented in Nigeria’s Federal Capital Territory (FCT), Abuja and 8 states of the federation; Anambra, Bauchi, Bayelsa, Ebonyi, Kaduna, Niger, Ogun, and Zamfara – for a geographic spread.

With an annual budget of over N100,000,000 NGN (USD 602,410), the scheme was designed to incentivize enrolled beneficiaries who fulfil a set of four co-responsibilities along the continuum of care for MNCH services in designated PHCs. The incentives was initiated to reduce the impact of economic barriers to accessing health services e.g. transportation to the PHC and out-of-pocket expenses [13]. Drawing from findings of the proof-of-concept phase [18], the value of N 5,000 or USD30 as at year 2012 was determined by average estimates of costs incurred by pregnant women to access and utilize services at health facility. Hence, pro-rated cash support of up to N5,000 (approximately USD 30) was provided to qualified pregnant women who go through the continuum of care, from antenatal through post-natal care. As at December 2014, the programme enrolled over 40,000 beneficiaries (pregnant women) and disbursed about N108,330,635.00 in the eight pilot states plus the FCT-Abuja.

It is worth noting, that in recent times (2015-2018), Nigeria government rolled out a new cash transfer programme which paid out N5000 (USD30) cash support to 40,000 poor Nigerians, selected from 20 states under the National Social Safety Nets Project (NASSP) in the presidency [19]. However, it is not clear whether lessons learned or recommendations from previous CCTs in the country have been considered during the design and implementation of the ongoing CCT programmes. Overwhelming evidence have evaluated the impact of CCT schemes around the world however, some contest whether they actually alleviate poverty as intended or whether they are just there to promote positive behavioral change [8, 20]. Countries in Latin America have predominantly provided evidence on reviews of CCT schemes effectiveness and impact on health and findings from these reviews come with limited transferability to the social, cultural, and political environments in sub-Saharan Africa [21, 22]. Yet, studies that assess the effectiveness of the design and implementation of CCT schemes in low-resource settings remain conspicuously lacking [20]. For global health interventions to increase their likelihood for success, scale, and sustainability, systematic insights on how implementers achieve success, or not; what problems were successfully addressed, or not; or how situational variability affected successes and challenges must be examined [8]. Hence, our study independently assessed the effectiveness of the day to day operations of the SURE-P MCH CCT programme in Nigeria to present useful insights into the design and implementation of the scheme to help policymakers improve on future CCTs interventions.

### Description of the SURE-P MCH CCT pilot Programme

The partial removal of fuel subsidies in January 2012 by Nigeria’s government, culminated in the establishment of the Subsidy Reinvestment and Empowerment Programme (SURE–P) to efficiently manage all financial resources accruable from the policy [23]. The Federal Government’s portion of the subsidy savings were invested in social safety net interventions as well as infrastructural and human development programmes to stimulate Nigeria’s economy and alleviate poverty. With a focus on accelerating progress on the fourth and fifth Millennium Development Goals (MDGs), the Federal Government of Nigeria invested part of the subsidy savings in improving maternal and child survival nationwide through the SURE-P Maternal and Child Health (MCH) [17]. This component of the SURE-P was implemented through a programme management unit (PIU) with headquarters in FCT-Abuja under the National Primary Health Care Development Agency and was premised upon the already existing Midwives Service Scheme (MSS) of the agency [24]. The MSS facilitated the deployment and redistribution of midwives to selected primary healthcare centres across the country to increase access to quality maternal and child health services [25]. However, the SURE-P MCH expanded the benefits of MSS by focusing on supply and demand sides interventions while creating new strategies to reach the most vulnerable and underserved populations in Nigeria with access to basic MNCH services.

Through the SURE-P MCH supply side interventions, trained midwives and Community Health Extension Workers (CHEWs) were deployed to designated facilities across Nigeria’s 36 states and the FCT, and skills sets of existing cadres of health staff across health facilities were strengthened [25]. MNCH medicines, consumables and equipment were supplied and infrastructural developments such as renovation of primary health centres and provision of bore holes to enhance access to potable water were put in place. On the demand creation interventions, SURE-P MCH utilized the conditional cash transfer (CCT) programme as a key demand booster for MNCH services to complement the huge supply-side interventions [26]. Nevertheless, the SURE-P MCH CCT programme was introduced to check whether the added cash incentive would have a significant impact on the utilization of ante-natal care services and facility-based childbirth among pregnant women in SURE-P MCH target communities. Following a six-month proof of concept phase where the CCT concept and design were fine-tuned in Kuje PHC and Karu PHC, both in FCT –Abuja, the SURE-P MCH CCT pilot programme was officially launched on May 13, 2013 at Deidei Comprehensive Health Centre Bwari, Bwari Area Council Abuja-FCT. A cluster model was used to select four (4) Primary Health Care (PHC) facilities per state with the exception of FCT-ABUJA which had five (5) PHC facilities making a total of 37 participating primary health care (PHC) facilities in 10 CCT clusters [27, 28].

### Design of the SURE-P MCH CCT Programme

#### Programme Beneficiaries

The primary beneficiaries of the CCT Programme were pregnant women who enrolled at designated SURE-P MCH CCT PHCs. However, secondary beneficiaries included their newborns, wider household (other existing children), as mothers contact with health services increased over the period and their husbands were relieved of some out-of-pocket expenses.

#### Eligibility

**Fig. 1:**
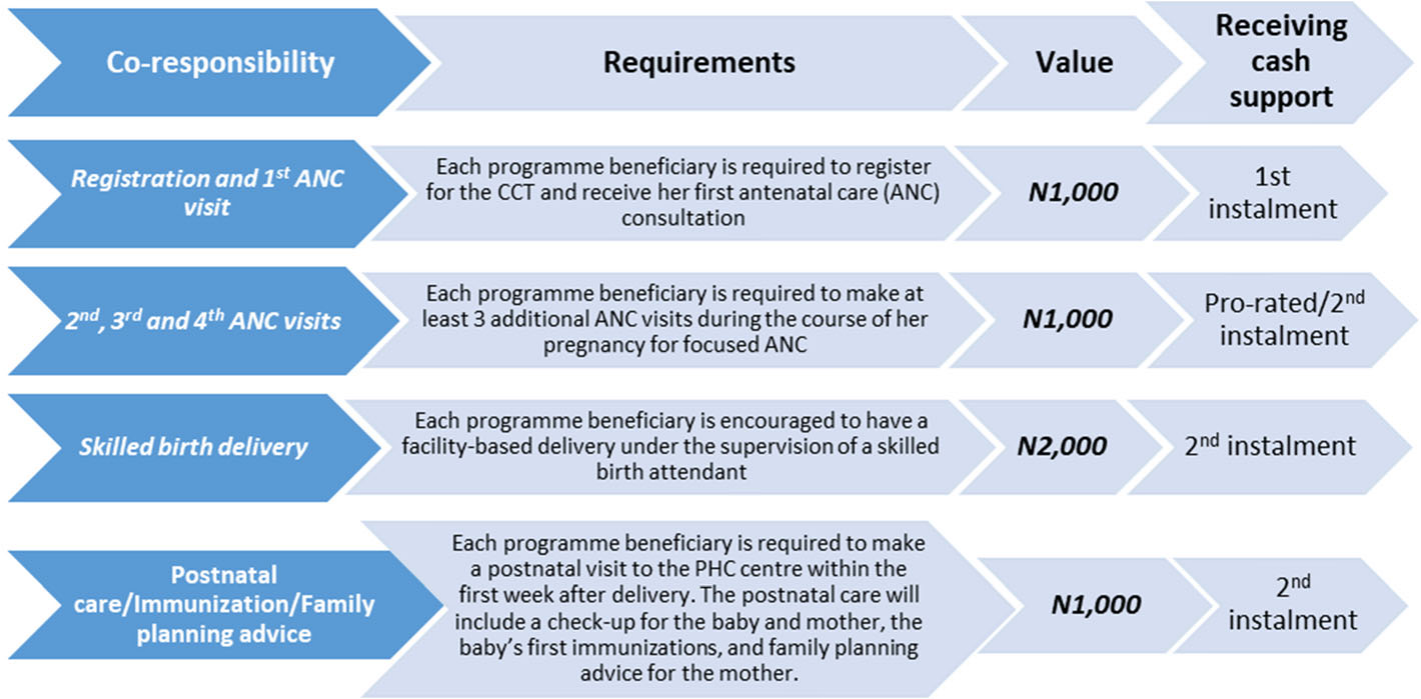
SURE-P MCH CCT Co-Responsibilities and Benefits

All pregnant women whose pregnancy status were confirmed and booked in each participating facility and who were yet to benefit from the CCT program met the eligibility criteria. The CCT Pilot programme incentivized beneficiaries with a total of N5000 (approximately USD30) cash support after fulfilling four set of co-responsibilities as detailed in Fig. 1.

#### Cash Transfers

The cash disbursement was designed to be done in two tranches, where each woman receives the first disbursement of N1,000 after registration at a participating PHC. Then the second and final disbursement up to N4,000 according to the co-responsibilities completed for ANC, childbirth with skilled birth attendant, postnatal care, neonate immunization, and family planning advice.

#### Referral to General Hospital

Following the design of the CCT programme, beneficiaries who enrolled in the CCT programme were entitled to free care at secondary health facility (general hospital) in their cluster whenever they are referred for elective or emergency obstetric treatment from their PHC.

#### Programme Administration and Operations

Prior to the CCT implementation, a two-week State Readiness Assessment (SRA) was conducted across all participating states to create awareness and sensitize various stakeholders at all levels on the SURE-P MCH CCT pilot programme and implementation plans. In each state, one CCT Technical Officer, one Field Supervisor and two Field Officers were recruited to manage the CCT implementation. A State Steering Committee (SSC) comprising of the Honourable Commissioner State Ministry of Health, Ministry of Women Affairs, Ministry of Local Government and Chieftaincy Affairs including Traditional and Religious Leaders, State Director Primary Health Care, State MDGs Focal Person, MSS Focal Person, and SURE-P State CCT Technical Officer was inaugurated to guide the implementation of CCT in each state to ensure strong stakeholder collaboration.

#### Training

Prior to CCT enrollment and registration of beneficiaries, service providers (Midwives, CHEWs and VHWs) were trained on programme implementation and the CCT reporting tools. Also, Ward Development Committee (WDC) members in all designated PHCs who were either re-activated or newly formed were given an orientation on the programme processes and what their responsibilities would be. The CCT reporting tools including; Beneficiary Registration Card, CCT Facility Registers, CCT Personal Consultation Forms and CCT Referral Forms were developed and deployed to all participating health facilities prior registration and enrollment of pregnant women.

#### CCT pay–roll processes

Pay-rolls were usually generated from a spreadsheet containing list of eligible beneficiaries, their registration numbers and the amount of cash support they qualify for, based on pre-conditions met. Prior to pay-out events, SSC, WDC and Officer-in-charge (OICs) of health facilities were duly informed about the dates. The pay-roll containing the payment schedule of beneficiaries was usually displayed at the notice board of the health facility to notify eligible beneficiaries about their qualification to receive the cash incentives. Additionally, the CCT technical staff would contact all qualified beneficiaries by telephone and inform them about the proposed pay-out day and also mobilize them for the event. CCT Pay-out information was also sent to the community through the village health workers (VHW) who inform the women about the date and time for the cash disbursements. The pay-roll was used on pay-out days to invite beneficiaries for their cash disbursements.

#### Pay-out events

The CCT pay-out event was the climax of the CCT programme because that was the day beneficiaries received their cash disbursements and was usually a one-day event but with possible spill-over into the next day. The pay-out event venues were usually organized with different stations in a linear manner to allow benefiting women move in a chronological manner for accreditation and validation before receiving their cash support. Thereafter, the CCT beneficiary receives their accrued cash support from bank cashiers who were present at the venues and being supported by the CCT operational staff. When a woman receives a total of N5000 (USD30) cash support having fulfilled all four co-responsibilities, the CCT registration card was retrieved from her and archived with the project.

## Methods

### Study design

The study was a combination of a desk review of SURE-P MCH CCT operational documents and a descriptive cross-sectional, retrospective survey of CCT primary beneficiaries using face-to-face interviewer administered questionnaires to elicit responses. The desk review understudied the SURE-P MCH programme implementation manual (PIM), and several SURE-P MCH CCT monthly reports obtained from the project management unit (PIU) while a structured questionnaire of (16) questions was used for the face-to-face interviews with CCT primary beneficiaries. At the end of the desk review and interviews, a Strengths, Weaknesses, Opportunities and Threats (SWOT) analysis was applied to the study findings. This helped to identify key strengths and weaknesses of the CCT scheme as well as opportunities and threats in order to identify key challenges and provide recommendations for future design and implementation of CCT Programmes.

### Study population

Study population was drawn from 37 participating CCT pilot PHC facilities in FCT-Abuja and eight (8) participating states of Anambra, Bauchi, Bayelsa, Ebonyi, Kaduna, Niger, Ogun, and Zamfara. However, following security and accessibility challenges, assessors were unable to reach all four (4) facilities in some states like Bauchi, Kaduna, Niger, Ogun and Zamfara, hence respondents were drawn from only 29 PHC facilities out of the 37 participating facilities. Respondents were pregnant women or nursing mothers who enrolled in the CCT programme during the pilot period from April, 2013 to May 2015 and who received cash incentives for meeting any of the four co-responsibilities.

### Sampling

A list of all women who enrolled in the pilot programme with their telephone contacts served as the sampling frame from which a table of random numbers generated with the Mersenne-Twister system was used to recruit participants into the study and participants who could not be reached on phone were replaced from the sampling frame by simply repeating the table of random numbers. The sampling frame consisted of all 37737 CCT pilot enrollees (Table 1).

**Table 1:** CCT Pilot Enrollees by State

State	No. of Enrollees
Anambra	1425
Bauchi	4120
Bayelsa	2571
Ebonyi	2752
FCT	16390
Kaduna	2733
Niger	2168
Ogun	1435
Zamfara	4143

A total of 314 CCT primary beneficiaries were selected using a two-stage sampling technique and 30–33 respondents were allotted to each state and FCT-Abuja. The decision on the sample size for each pilot state was made on the strength of the assumptions of the central limit theorem and resources available for the assessment.

### Data collection process

Data were collected between June–July 2015 (period when the CCT scheme had already ended) and started with the recruitment of Study Assessors who were trained on the assessment’s purpose, scope and objectives, interview process, administration of data collection instrument and techniques for conducting effective focus group discussions. Demographic data was collected on sex, age, religion, ethnic group, marital status, educational status, employment status, number of children and number of pregnancy. Assessors combined English with local languages to elicit responses from participants where and when necessary.

### Data management and analysis

Each of the answered questionnaires was coded and entered into a spreadsheet. The data collected was subjected to descriptive (i.e. mean, median and mode) and inferential (i.e. Chi-square) statistical treatment. Bivariate analysis and test of statistical significance were carried out using R version 3.1.2. [29] Data from respondents were grouped into themes for analysis as follows; CCT operations, CCTs pay- roll processes, CCTs pay- out events and perceived perception by beneficiaries. Finally, information obtained was summarized and presented in tables, charts and frequencies.

### Validity and reliability checks

The data collection instruments were put through phased validity which involved pre-testing and field testing of the tools. First the tools were pre-tested and peer-reviewed by internal and external colleagues to ensure internal consistency and validity. Then, a field testing of the questionnaires and survey methodology was done in one of the non-participating CCT facilities in FCT-Abuja with all Assessors who were part of the assessment training. Findings from the field-testing were incorporated and amended accordingly before commencing data colllection.

## Results

### Demographics of beneficiary survey

Thirty one and a half percent (31.5%) of beneficiaries were unemployed (i.e. housewives), 30.5% traders and 75% had a personal income equal to or less than N10,000 (USD60) monthly

### CCT operations

**Fig. 2:**
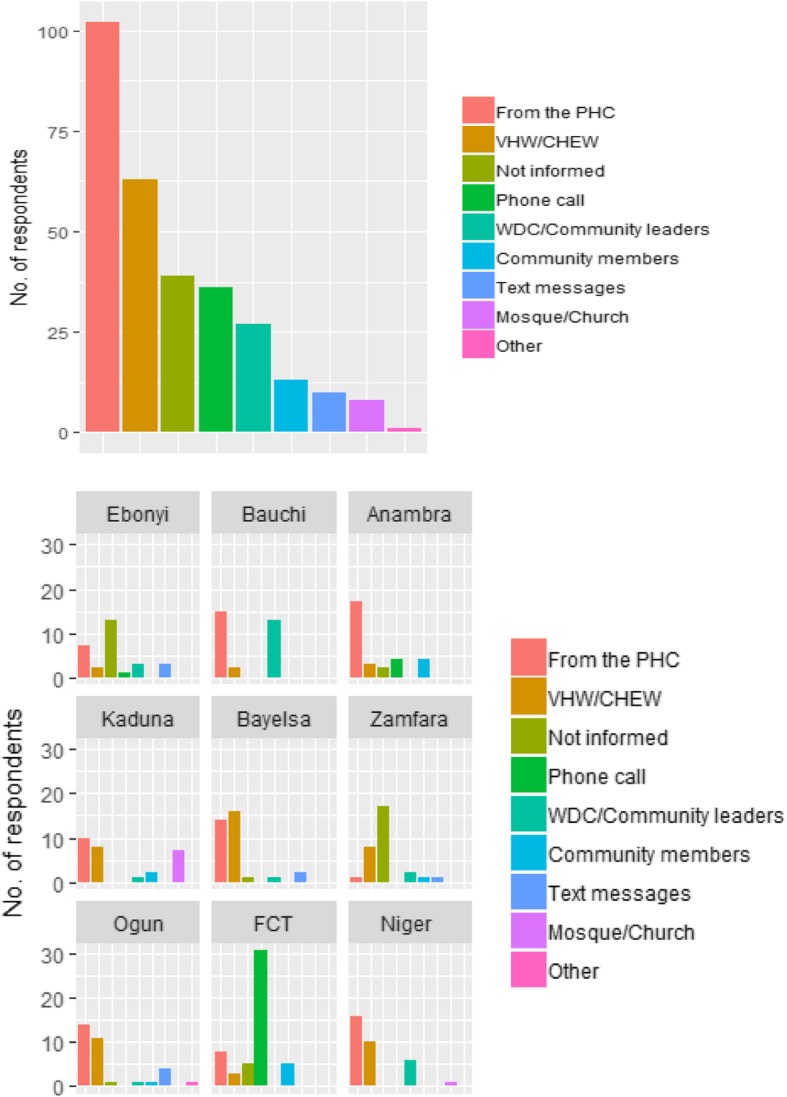
Where Beneficiaries first heard about CCT

About 22.6% of beneficiaries reported that they heard about the CCT programme from family and friends, while 43% heard from village health workers (VHWs), 18.2% heard from community health extension workers (CHEWs) and 11.1% heard from ward development committee (WDC) members as shown in Fig. 2. The data was statistically significantly across the various pilot states (*p* < 0.001).

Precisely 69.9% of respondents reported that they did not pay for routine PHC services, while another 30.1% admitted paying for PHC services. The services that was mostly paid for was routine ANC medicines (61.1% of respondents).

Most beneficiaries were enrolled in the pilot within the first 2 trimesters of their pregnancies (93%, Table 2), adequate information on their co-responsibilities was given to them at enrolment and they generally attested to the regularity of their CCT records.

**Table 2:** Pregnancy Stage at CCT Enrolment

Stage at Enrolment	No.	%
First trimester	122	40.00
Second trimester	163	53.44
Third trimester	19	6.23
At childbirth	1	0.33

Eighty six percent (86%) of respondents stated that they had no complaints about the programme at enrolment while majority of the women (92.28%) reported finding the CCT process to be easy – from enrolment to pay-out (Table 3).

**Table 3:** Perception of CCT Process

Perception	Freq	%
Easy	287	92.28
Not so easy	20	6.43
Difficult	1	0.32
Very difficult	3	0.96

### CCT pay-roll and payout events

Local health system structures played a more prominent role in disseminating information on Pay-Out Events with the women being mostly informed via the health facility (34.2%), VHW/CHEW (21.1%) and WDC (9.1%). Significantly, the phone calls from SURE-P CCT staff were prominent sources of information, according to about 12.1% of the respondents. 95% of the respondents said that the information they received was timely, while 84.8% said that the pay-outs were held as scheduled. In Niger State, most of them complained that the Pay-Out Events did not hold as scheduled (Table 4 & Table 5).

**Table 4:** How Beneficiaries Heard About Pay-Out Events

Source	No.	%
From the PHC	102	34.11
VHW/CHEW	63	21.07
Not informed	39	13.04
Phone call	36	12.04
WDC/Community leaders	27	9.03
Community members	13	4.35
Text messages	10	3.34
Mosque/Church	8	2.68
Other	1	0.33

**Table 5:** Were Pay-Outs According To Schedule?

State	Yes	No	Don’t know
Ebonyi	16	2	0
Bauchi	30	0	0
Anambra	28	0	0
Kaduna	25	3	0
Bayelsa	34	0	0
Zamfara	12	5	11
Ogun	31	1	0
FCT	47	0	0
Niger	12	20	0

Pay-out events were largely perceived by the beneficiaries to be orderly and well-organised (65.2%, *p* < .001); most complaints about the way Pay-Out events were organized came from Bauchi, Ebonyi, and FCT, but statistical significance was not tested because some of the assumptions for the sample could not be met (Table 6).

**Table 6:** Description of Pay-Out Events in Pilot States

State	Well organised	Fairly organised	Not organised
Ebonyi	11	1	2
Bauchi	2	20	5
Anambra	24	1	0
Kaduna	22	5	1
Bayelsa	33	0	1
Zamfara	12	0	0
Ogun	30	2	0
FCT	23	17	7
Niger	8	25	1

Most of the women said that they were paid the complete N5,000 incentive (39.8%), followed by those who received only N1,000 (28.1%). Most of the respondents who received the incentive were paid in a single instalment.

There was a statistically significant variation (*p* < 0.001) amongst the CCT Pilot States of the amount received by the beneficiaries; the highest proportion of those that collected N5,000.00 were from FCT (30.4%) while 38.4% of those that collected only N1,000.00, were from Niger State. It should be noted that the CCT Pilot Programme started in the FCT several months before the other States.

There was also a statistically significant variation (*p* < 0.001) amongst Pilot States of the amount received by the beneficiaries; the highest number of those that collected N5,000.00 were from FCT, followed by Ogun State. Of the beneficiaries that collected only N1,000.00, most of them were in Niger State.

**Fig. 3:**
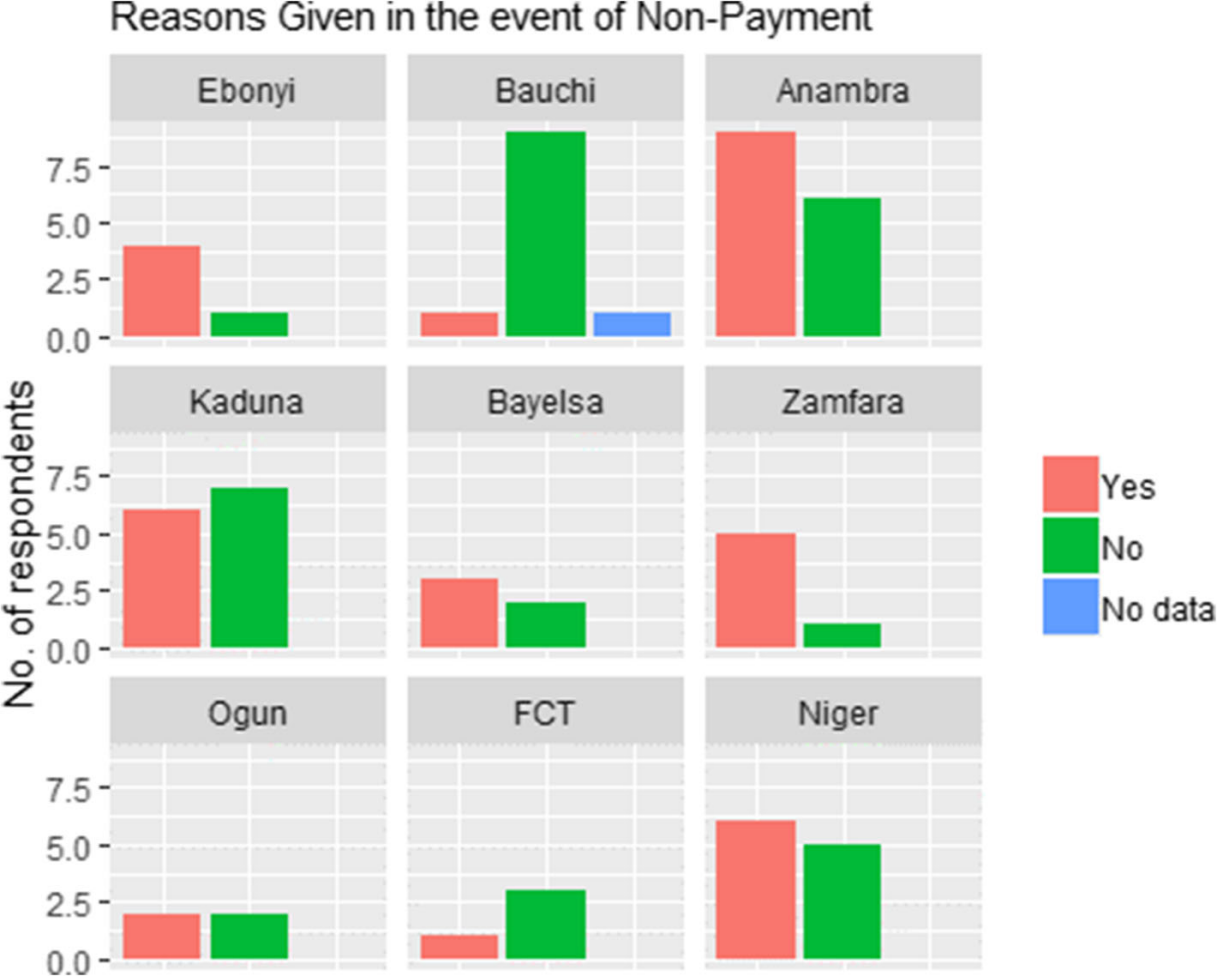
Beneficiaries Given Reasons for Non-Payment

Out of the CCT enrollees that were not given any money, about an equal number were or were not given any reasons why they were not paid (50.7 and 49.3%, respectively). When analysed on a State-by-State basis, 90, 75 and 53% of the respondents in Bauchi, FCT and Kaduna Pilot States, respectively, said they were not given any reasons for non-payment. In contrast, beneficiaries from Zamfara, Ebonyi and Anambra Pilot States scored CCT high (83.3, 80 and 60%) in conveying to them any reason for their non-payment (Fig. 3).

## Discussion

Social transfers such as CCTs are usually associated with various errors including design and implementation, administrative costs and other secondary consequences [30] however, the SURE-P MCH CCT was designed with novel approaches drawn from similar schemes around the world and lessons from a prior proof of concept phase, yet its implementation was more complex than its design [27]. Several challenges such as omission of eligible participants’ names from the pay-roll and irregularities on the pay-roll negatively affected the pay-roll processes and resulted in irregular pay-out events. The irregular pay-out events were reported to be as a result of irregular availability of funds from the federal government to the PIU, making the CCT programme to deviate from its initial design of two installments for each woman to operationally making single disbursements during implementation. The initial payment design was to pay every woman, first after registration and secondly after meeting the remaining set of co-responsibilities disbursements. This deviation in cash disbursements to single payment resulted in combining several beneficiaries eligible for payments to receive their cash transfers in one installment and this obviously led to the overcrowding and long waiting hours experienced at pay-out events. The single payment thwarted the essence for the design which was to make payments prompt, build beneficiaries’ trust and for the cash to serve its intended purpose of reducing healthcare costs for mothers [18].

Furthermore, beneficiaries who qualified for cash disbursements reported that they were mostly informed about the pay-out events via the health facility (34.2%), VHW/CHEW (21.1%) and WDC (9.1%) and this is consistent with previous findings that local health system structures play a more prominent role in disseminating information on Pay-Out Events [32]. Significantly, the phone calls from SURE-P MCH CCT Officers were also prominent sources of information, according to about 12.1% of the respondents and 95% of the respondents stated that the information they received was timely. Unfortunately, the desk review identified that beneficiaries who were entitled to free care at the general hospital when referred from their PHC for elective or emergency obstetric treatment faced a lot of hitches as beneficiaries lamented their frustrations in assessing that level of care. Owing to programmatic challenges [Table 7], none of the women who were referred due to obstetric complications benefited from the referral service as designed for the scheme.

**Table 7:** Key challenges

1. Irregular and Inconsistent Pay-Out Events: The CCT pay-out events were highly inconsistent and irregular resulting in large crowds which posed a big challenge for crowd control and management. Owing to the large crowds, women were kept for longer than necessary just to receive their cash support. It was gathered that this problem was as a result of delay in releasing funds to the SURE P MCH Programme Implementation Unit.	
2. Security for Cash Disbursements: Adequate security was not provided for the CCT pay-out events and this compromised the safety of the cash being disbursed and placed paying staff at high risk.	
3. Non-Payment of Cash Incentives: Several of the women interviewed reported not being paid their entitlement and others who received their first instalment were yet to receive their second instalment. It was later confirmed that the SURE-P MCH PIU had recently received funds and was in the process of conducting another round of CCT pay-out events in the states.	
4. State Steering Committees: The SSC meetings were not sustainable as a result of unavailability of imprest to fund such meetings and this impacted negatively on the SSC as it never really carried out its assigned role.	
5. Monitoring and Evaluation: Monitoring of the CCT Pay-out events were limited due to a lack of an M&E plan which delayed relevant programmatic interventions when needed.	
6. Additional workload: The additional demand for MNCH services generated by the CCT Programme in the facilities created additional workload for the participating facilities, particularly the obligation to complete the CCT reporting tools which had varying levels of capacity to handle the work.	
7. Referrals: Initially the CCT Programme was designed to take care of women who had complications and had to be referred to General Hospitals but this strategy was marred with a lot of hitches as beneficiaries lamented their frustrations in assessing that level of care.	

Monitoring programme uptake and performance in each implementing cluster is essential to track and address programmatic challenges which may be the result of operational barriers [18]. However, the CCT pay-outs lacked a clear monitoring framework that would have helped the programme to make necessary process amendments to improve outcome of the pay-out events. This conspicuous lack of (M&E) mechanisms is similar to COPE’s implementation which was also inundated with several service delivery challenges that undermined programme delivery and effectiveness [11]. The use of a cash and paper based payment system to track and pay clients, [32] heightened the need for adequate security for the cash and staff involved for the pay-out events and also heightened loophole for corruption or fraud to thrive. This also indicates that implementing a CCT programme involves huge administrative and management costs as several staff are usually needed to successfully execute activities at the different levels of the process.

Hence, the effectiveness of the day to day operational processes of the SURE-P MCH was obviously challenged. However, despite the implementation challenges, 86% of respondents stated that they had no complaints about the CCT programme at enrolment while another majority (88.7%) opined that the CCT operational processes were easy, from enrolment to pay-out. Though respondents reported perceived satisfaction with operational processes however, there was still wide spread complaints about the pay-out events which was usually overcrowded resulting in long waiting time for beneficiaries. This twist by respondents corroborates earlier studies, which documented patient’s under-reporting of poor experiences [31]. Finally, most respondents expressed satisfaction with the operational processes having perceived the scheme as a successful government programme that should not be discontinued.

### Limitations of study

This study was limited by the paucity of literature on previous CCTs in Nigeria to support the desk review, though all literature available was considered. Also, the inability to assess more beneficiaries from all 37 facilities following security issues and inaccessibility of some terrain were limiting. However, the 29 facilities and 314 beneficiaries assessed still gave a fair assessment of all participating states covered.

## Conclusion

The major strength of the SURE-P MCH CCT was its design to complement other supply-side interventions as a motivator to enhance access to health facilities where free services were provided. Evidence shows that for a demand-side intervention such as CCT to be successful, concurrent supply-side inputs should be available [33]. Despite demonstrating a robust design of the CCT programme, its operational processes were marred with numerous challenges including: delay in cash disbursements to qualified beneficiaries owing to delayed replenishment from the federal government; omission of eligible participants names from the pay-roll; irregularities on the pay-roll; over-crowding of pay-out events leading to long waiting times and lack of access to referral facilities in case of emergencies or obstetric complications. The 93% enrollment recorded within first 2 trimesters indicates that the CCT programme encouraged women to register and attend antenatal visit (ANC) and Skilled Birth delivery hence, motivating positive behavior change towards facility use among beneficiaries and their communities [3, 34]. Key challenges that marred the operational effectiveness of the scheme and key recommendations to mitigate such challenges in the future are identified in this paper. Future CCT programmes should understudy the SWOT analysis [Table 8] to guide future interventions in Nigeria and beyond.

**Table 8:** Strength, Weaknesses, Opportunities, Threat (SWOT) Analysis

Strengths	Weaknesses
Improved Health Facility attendance: 93% enrollment within first 2 trimesters indicates that the CCT programme encouraged women to register and attend antenatal visit (ANC) and Skilled Attendance during childbirth. Early results from the CCT Programme pilot phase was highly positive in terms of before-and-after comparisons of facility attendance in the selected clusters, and feedback from facility staff and women accessing the programme [18].	Irregular and Inconsistent pay out events: The pay-outs were highly inconsistent and irregular following delayed release of funds from federal government. This resulted in large crowds during pay-out events and long waiting times for beneficiaries.
Supply-side intervention: The combination of the CCT programme with supply-side interventions where 69.9% of respondents reported not paying for PHC services motivated more women to seek health services and utilize other free medical services provided at SURE-P supported PHCs.	Non-Payment of Cash Incentives in two installments: Some respondents reported that they were not paid their full entitlement. Only 39.8% of eligible beneficiaries received the full N5,000. Many others who received their first installment were yet to receive the second installment.
Robust design of the CCT programme: The desk review showed that the CCT was designed with novel approaches drawn from similar schemes around the world. Adequately conceptualized strategic plan and implementation manual, clear co-responsibilities for eligibility, two installment payment plans to facilitate prompt payments, etc.	Referral to General Hospital: Beneficiaries with complications were unable to access the services of general hospitals at no cost, owing to programmatic challenges (Table 8). The referral loop was problematic and deprived beneficiaries from utilizing the service.
Beneficiary retention: The CCT Programme contributed immensely in sustaining beneficiary retention throughout the continuum of care as about 88.7% of respondents followed the programme from enrolment to pay-out and fulfilled their four co-responsibilities.	State Steering Committees (SSC): The desk review showed that the SSC meetings were irregular. However, as a result of unavailability of imprest account to fund such meetings, the SSC may not be sustainable.
Staffing and Capacity Building: The CCT Programme was adequately staffed at all levels from the PIU to field offices and all stakeholders were trained prior to the implementation. This facilitated the smooth roll-out of the programme and contributed to the 86% respondents who had no complaints about the CCT programme at enrolment and 88.7% who found the operational process to be easy – from enrolment to pay-out.	Security: The desk review indicated that security during CCT pay-out events was inadequate and this raised a lot of security concerns for the cash being disbursed at the venue by officers that make the payments.
Documentation and reporting tools: The timely provision of the CCT reporting tools such as; Beneficiary Registration Card, CCT Facility Registers, CCT Personal Consultation Forms and CCT Referral Forms CCT Tools, prior to registration and enrollment of pregnant women helped seamless documentation and easy information retrieval.	M & E Framework: There was no clear M and E framework for the CCT programme and particularly for the Pay-out events and this resulted in non-implementation of programmatic recommendations.
Opportunities	Threats
CCT awareness increased patronage for the PHCs: Respondents reported hearing about the CCT from family and friends, village health workers (VHWs), community health extension workers (CHEWs) and ward development committee (WDC) members. This led to a massive awareness of the CCT programme within the communities and huge patronage for the primary health facilities.	Lack of a sustainability plan: The CCT intervention was designed as a social safety net program without an apparent sustainability plan.
Availability of Skilled Health work force at the PHCs: The supply side component of the SURE-P MCH provided trained midwives and Community Health Extension Workers (CHEWs) at the PHCs who offered quality care to CCT enrollees and stimulated the smooth operation of the CCT operations.	Unsustainability of the SURE-P MCH Programme: Continuation of the CCT programme was largely dependent on the SURE-P MCH project which was politically motivated, therefore a regime change threatened its continuation.
Availability of other supply side incentives such as ‘Mama Kits’ which contained delivery items for mother and baby, free medicines etc. further attracted women to register for the CCT.	Unavailability of CCT funds at the PIU: Funds budgeted for CCT pay-outs were largely unavailable from the federal government on schedule. This hampered the funds disbursement plans.
Huge Potential: The design of the CCT Programme to support other supply side interventions helped entrench health seeking behaviours within communities.	Discontinuation: A sudden discontinuation of the programme resulted in thwarting the numerous gains made and reversed the positive trends achieved so far.
Access to health services: The use of the CCT to boost demand for MNCH services created more space for clients’ interaction with service providers at the health facilities.	Distrust for Government: Stoppage of the CCT programme led to strong distrust/lack of confidence for government programmes.

### Key recommendations

1. **CCT Payment Method**: A prudent and effective payment method that delivers quick and prompt payments should be considered to check time wastage, avoid fraud, build trust and simplify the process for pregnant women and nursing mothers.
2. **Regular and Consistent Payment Schedule**: There should be a standard protocol/guideline for CCT pay-out events. At least, the women should be paid every quarter to shorten the long wait for payments by beneficiaries and restore their confidence in the system. This means that whatever funds earmarked for CCT from inception should be made available to the implementing unit in a timely manner to ensure effective implementation.
3. **Monitoring and Evaluation**: There should be strong monitoring of the CCT Pay-out events to address immediate programmatic gaps during implementation through a robust M&E plan.
4. **Auditing**: Periodic auditing and independent evaluation of the CCT registers will enhance compliance and adherence to the set conditions. It will reduce the chances of fraud and enhance the overall credibility of the pay-out system.
5. **Exploring other forms of incentives**: As CCT significantly improved demand for health intervention/services in the communities, respondents for other forms of incentives which could provide long term benefits to beneficiaries e.g. (vocational training/skill acquisition incentives). The CCT Programme should also be weighed with cash-benefit induced pregnancies vis-á-vis other forms of incentives.
6. **Involvement of PHC staff in cash-disbursements**: This can be considered as it strongly came out in the recommendations from the field that the use of facility management staff for cash disbursement will strengthen the disbursement process.
7. **Financing Mechanism and Programme Continuation**: Long- term financing mechanisms should be considered for CCT interventions in the country by enacting public policies that will guide the conceptualization and funding of future CCT programs.

## Data Availability

The data and materials for this assessment are available from the corresponding author on request.

## Abbreviations

ANC: Antenatal care
CCT: Conditional cash transfer
CHEWs: Community health extension workers
COPE: Care of the People
FCT: Federal capital territory
FGN: Federal Government of Nigeria
M & E: Monitoring and evaluation
MCH: Maternal and Child Health
MDG: Millennium development goals
MNCH: Maternal, neonatal and child health
MSS: Midwives service scheme
PHC: Primary healthcare facility
PIM: programme implementation manual
PIU: Project implementation unit
R* Statistical package: R is an open source statistical package developed by The R Foundation for Statistical Computing
SBA: Skilled birth attendance
SRA: State Readiness Assessment
SSC: State Steering Committee
SURE-P: Subsidy Reinvestment and Empowerment Programme
SWOT: Strength, weaknesses, opportunities and threats
USD: United States Dollars
VHW: Village health worker
WDC: Ward Development Committee
